# DigiNet: Optimizing personalized care for patients with stage IV non-small cell lung cancer (NSCLC) through a digitally connected provider network–analysis plan of a prospective multicenter cohort trial

**DOI:** 10.1007/s00432-025-06275-x

**Published:** 2025-09-09

**Authors:** Anika Kästner, Anna Kron, Leonie Eilers, Anna Spier, Vanessa Mildenberger, Dusan Simic, Stephanie Stock, Florian Kron, Matthias Scheffler, Gerhard Schillinger, Neeltje van den Berg, Jürgen Wolf, Wolfgang Hoffmann

**Affiliations:** 1 Institute for Community Medicine, Section Epidemiology of Health Care and Community Health, University Medicine Greifswald, Greifswald, Germany; 2 National Network Genomic Medicine, University Hospital of Cologne, Cologne, Germany; 3 Department I of Internal Medicine, Center for Integrated Oncology Aachen Bonn Cologne Duesseldorf, Lung Cancer Group Cologne, University Hospital of Cologne, Cologne, Germany; 4 FOM University of Applied Sciences, Essen, Germany; 5 Institute of Health Economics and Clinical Epidemiology, University of Cologne, Faculty of Medicine and University Hospital of Cologne, Cologne, Germany; 6 Federal Association of the AOK, Berlin, Germany

**Keywords:** Non-small cell lung cancer, Personalized medicine, Digital network, Quality of life, Cohort study, Real-world data

## Abstract

**Purpose:**

The German sector-based healthcare system poses a major challenge to continuous patient monitoring and long-term follow-up, both essential for generating high-quality, longitudinal real-world data. The national Network for Genomic Medicine (nNGM) bridges the inpatient and outpatient care sectors to provide comprehensive molecular diagnostics and personalized treatment for non-small cell lung cancer (NSCLC) patients in Germany. Building on the established nNGM infrastructure, the DigiNet study aims to evaluate the impact of digitally integrated, personalized care on overall survival (OS) and the optimization of treatment pathways, compared to routine care.

**Methods:**

DigiNet is a prospective, controlled, non-randomized multicenter cohort study including patients with stage IV NSCLC in two study regions (East and West) in Germany. The results of molecular diagnostics and clinical information, along with the entire treatment data are documented in a shared database. A board of lung cancer specialists monitors critical events. Patients digitally complete quality of life questionnaires, with results visualized for physicians. To assess the impact of this personalized digital care, a population-based control group will be identified by matching cohorts within the involved cancer registries. The primary endpoint is OS, and secondary endpoints comprise time on first-line treatment and hospitalization rates. Furthermore, a health economic and business economic evaluation will be conducted. Qualitative interviews with patients and physicians will be performed to assess barriers and facilitating factors for implementing the DigiNet intervention.

**Ethics:**

The study protocol was reviewed and approved by the Ethics Committee of the University Hospital of Cologne (21-1521).

**Trial Registration:**

NCT05818449, registered retrospectively on December 12, 2022.

**Supplementary Information:**

The online version contains supplementary material available at 10.1007/s00432-025-06275-x.

## Introduction

As lung cancer remains one of the most incident and deadliest malignancy worldwide, with the highest economic burden, treatment within well-organized clinical networks aims to generate evidence and improve patient outcomes (Chen et al. 2023; Luengo-Fernandez et al. 2013; Sung et al. 2021). The national Network Genomic Medicine (nNGM) Lung Cancer was established based on the concept that centralizing comprehensive molecular-pathological diagnostics and therapy recommendation from the time of initial diagnosis enhances efficiency, while decentralizing subsequent treatment ensures equitable and high-quality care for all patients across Germany. Established in 2018, the network currently comprises 29 highly specialized nNGM centers, offering harmonized molecular pathological diagnostics, interdisciplinary case assessments, standardized clinical information (including in-label or off-label therapies and clinical trial options), joint tumor boards, and ongoing evaluation. To support networking between inpatient and outpatient care sectors, the nNGM established a complex Network Data Platform (NDP) to integrate heterogeneous central and decentralized IT systems from nNGM centers and regional partners. This infrastructure enables continuous and multidirectional data exchange for both patient care and research within the nNGM. However, long-term patient follow-up after nNGM diagnostics and initial therapy remains a significant challenge, as patients often consult different healthcare providers.

Building on the comprehensive groundwork of nNGM, the DigiNet study was therefore designed as digital network to ensure continuous follow-up, ongoing coordinated treatment management, and real-world data collection throughout the entire patient journeys. By integrating healthcare providers along the individual treatment pathways, actively involving patients, and harmonizing the documentation of nNGM and DigiNet data, the study aims to establish a new care approach. Its primary objective is to evaluate the impact of this digitally integrated, personalized care on overall survival in patients with advanced NSCLC. The DigiNet study was designed for the two model regions East (comprising the German federal states of Berlin and Saxony) and West (primarily in the German federal state North Rhine-Westphalia). These regions were selected based on the presence of high-volume nNGM centers, defined by patient numbers and the strength of their regional partner networks, thereby ensuring sufficient sample sizes for effective implementation of the DigiNet intervention. The regional partners were selected in coordination with the German Cancer Society (DKG) and the Federal Association of Outpatient Hematologists and Oncologists (BNHO) to ensure successful recruitment. The DigiNet intervention group is recruited as a prospective single-arm cohort, while the population-based control group will be identified retrospectively through cohort matching using data from the respective state cancer registries. This registry-based design leverages Germany’s mandatory cancer reporting system, which ensures near-complete and standardized documentation of all advanced NSCLC cases, allowing for a representative population-based control group. A randomized design was not feasible, as DigiNet builds upon the long-established nNGM network structures, including numerous practitioners across inpatient and outpatient sectors. Randomization would have required fundamental changes to established care structures and disrupted existing clinical workflows. Moreover, it may have reduced acceptance among providers and patients, potentially introducing selection bias. This pragmatic, non-randomized design allows for timely implementation across care settings while preserving continuity of care, particularly in the complex and evolving treatment landscape of advanced NSCLC. Following a positive evaluation, a nationwide rollout on top of the nNGM and potential integration into routine care are planned.

Consequently, nNGM has evolved into a national program for precision medicine in lung cancer, with a focus on optimizing care, particularly in the areas of comprehensive molecular testing, therapeutic decision-making, and outcome evaluation. DigiNet is a pilot project within the nNGM framework, aiming to establish a digitally supported network as an innovative care model (“neue Versorgungsform”) for coordinated treatment across all sectors and along the entire patient journey, including the active involvement of patients themselves. The DigiNet pilot study is designed as a prospective cohort study, primarily comparing outcomes of DigiNet participants with those outside the nNGM, using claims data and regional cancer registries. By doing so, DigiNet contributes to key objectives defined by the Connected Care Working Group under Germany’s National Decade Against Cancer (“Die Nationale Dekade gegen Krebs” 2025).

## Methods and analysis

The DigiNet study is funded by the Innovation Fund of the Federal Joint Committee (Gemeinsamer Bundesausschuss, G-BA) in Germany (01NVF20021). The DigiNet study was first registered on ClinicalTrials.gov on December 12, 2022 (ClinicalTrials.gov ID: NCT05818449). The following protocol adheres to the SPIRIT 2013 Statement (Chan et al. 2013). The SPIRIT reporting checklist is provided in Supplementary Information file SI1.

### Primary aim and objective

The aim of the study is to optimize and enhance personalized cancer care (molecularly targeted therapies) for patients with advanced NSCLC by digitally connecting specialized nNGM centers with regional partners from hospitals, medical care centers, oncological practices, and the patients to promote intersectoral collaboration. The primary objective is to compare overall survival (OS) between advanced NSCLC patients receiving personalized, digitally assisted lung cancer care and a population-based control group receiving standard care.

### Study design

DigiNet is a prospective, controlled, non-randomized, non-blinded, multicenter cohort study conducted in two model regions East (comprising the German federal states of Berlin and Saxony) and West (in the German federal state North Rhine-Westphalia). The study duration is four years from September 01, 2021 to September 30, 2025.

In brief, the DigiNet intervention group is actively recruited as a single-arm cohort based on written informed consent. The population-based control group will then subsequently be identified by cohort matching within the federal state cancer registries of the model regions.

For sensitivity analyses, two further control groups will be differentiated: a nNGM control group (patients participating within nNGM during the DigiNet study period) and a historical nNGM control group (patients participating within nNGM before the DigiNet study period).

### Eligibility criteria

Patients with the first diagnosis of stage IV non-small cell lung cancer (NSCLC) are eligible for inclusion in the study. Patients with previous lower stages of NSCLC are eligible for the study at the time of stage IV cancer progression. Patients have to be at least 18 years old, with a life expectancy of at least four weeks at the time of recruitment. Patients with a lack of written informed consent, illnesses, and conditions that impair compliance (e.g., dementia, addictive disorders, psychosis), or a severely impaired general condition that no longer permits lung cancer treatment are excluded.

### Study timeline

The study duration is four years in total and is divided into the following phases:

- Study preparation phase (8 months): October 1, 2021–May 31, 2022
- Recruitment of the DigiNet intervention group (22 months): June 1, 2022–March 31, 2024
- Follow-up phase of the DigiNet intervention group (12 months): April 1, 2024–March 31, 2025
- Collection of control group & evaluation phase (6 months): April 1, 2025–September 30, 2025

No interim analyses are planned.

### Study setting and study groups

#### Cohort 1: DigiNet intervention group

The participating specialized nNGM centers in the model regions are the University Hospital of Cologne, University Hospital Essen, Charité—Universitätsmedizin Berlin, Helios Klinikum Emil von Behring, and University Hospital Carl Gustav Carus and Medical Faculty of the TU Dresden, which are digitally connected to regional hospitals, medical care centers and oncology practices in routine care. A list of all participating sites and physicians can be found at ClinicalTrials.gov (*ClinicalTrials.gov: Improvement of Personalized Lung Cancer Care Through Digital Connection and Patient Participation (DigiNet): NCT05818449*, 2024). The DigiNet intervention group is actively recruited by the participating physicians after obtaining written informed consent and receives the intervention as described below.

#### Cohort 2: population-based control group

The population-based control group is determined by cohort matching within the cancer registries of the model regions and includes patients who fulfill the inclusion criteria but do not participate in neither DigiNet nor nNGM (non-DigiNet and non-nNGM patients). No informed consent is obtained from this group.

#### Cohort 3: nNGM control group

The nNGM control group includes patients with stage IV NSCLC in the model regions who participate in nNGM during the DigiNet study period but not in the DigiNet study and who gave their consent to the use of data for research purposes. The baseline data are documented in the nNGM database and combined with data from the cancer registry, in particular with regard to the survival status.

#### Cohort 4: historical nNGM control group

This historical nNGM control group includes patients with a first diagnosis of stage IV NSCLC in the model regions who participated in nNGM before the DigiNet study period (February 2018 to August 2021) and who gave their consent to the use of data for research purposes. The patient data were collected within the nNGM database and merged with claims data of the AOK statutory health insurances participating in nNGM.

### DigiNet intervention

#### nNGM diagnostics and clinical information

Each DigiNet patient receives standardized molecular diagnostics based on NGS, which is performed at one of the participating nNGM network centers. The tumor material is comprehensively examined for all known therapeutically relevant driver mutations.

The participating DigiNet physicians then receive molecular-pathological results from the nNGM. To harmonize the interpretation of diagnostics results and provide consistent treatment options, a dedicated database (MURIEL/MURIPEDIA) was developed within nNGM. Expert annotations of molecular-pathological data are created by nNGM specialists within the centers and regularly updated to reflect medical advances. All nNGM physicians can access these annotations and clinical information via the MURIEL/MURIPEDIA database, where all updates and modifications to treatment options are also visible over time. The personalized nNGM report includes clinical information along with possible treatment options (MURIEL/MURIPEDIA harmonized data), which may include available in-label and off-label treatments, clinical trials, and recommendations for presentation to molecular tumor boards, depending on the individual findings. Then, the physicians or assistants document the results of the molecular diagnostics in an eCRF in the shared database (NDP).

#### Therapy monitoring

The DigiNet intervention includes close therapy monitoring, starting with the initiation of first-line treatment in stage IV NSCLC at baseline, based on the initial molecular-pathological results from the nNGM. Deviations from the MURIEL/MURIPEDIA clinical information for treatment are considered critical events and are evaluated by a board of lung cancer specialists (expert panel). These deviations must be documented and justified to ensure coordinated DigiNet patient management. Other critical events include incomplete molecular pathological testing, lack of re-biopsy in relapse under targeted therapy and discontinuation of all systemic therapies due to side effects. All therapy course and outcomes data are systematically recorded as visits (physician–patient contacts) and must be fully documented by the DigiNet physicians in the NDP. In case of a disease progression relevant to therapy monitoring, follow-up continues accordingly, e.g., by indicating the need for re-biopsy and a new nNGM report.

#### Patient-reported outcomes (PROs)

The DigiNet patients actively participate in the study by recording patient-reported outcomes (PROs) via a patient portal. The PROs will be assessed monthly throughout the study by the patients themselves or with the support of the study nurses. The questionnaires cover the areas of health-related QoL, symptom control, functional status, mobility, anxiety, and depression. Patients can complete them digitally via the NDP or on paper. The following standardized and validated questionnaires are implemented (Koller et al. 2020; Löwe et al. 2010):

- European Organization For Research And Treatment Of Cancer (EORTC) Quality of Life Questionnaire Core Module (QLQ-C30): questionnaire for assessing the quality of life of cancer patients
- EORTC lung cancer module (QLQ-LC29): questionnaire for assessing the quality of life of lung cancer patients
- Patient Health Questionnaire-4 (PHQ-4): ultra-short screening for anxiety and depression

The PROs are automatically scored according to the developers’ specifications. When filled out digitally, the results of each of the PRO scales are visualized via a separate dashboard in the NDP in real-time to the treating physician. The DigiNet intervention involves the treating physician monitoring this information during the follow-up visits and considering the findings in the differential therapy (e.g., the use of a concomitant medication or the initiation of physiotherapy or psychotherapy), providing complementary data that may impact the course of treatment.

In the context of the health economic evaluation, the EuroQol Group EQ-5D-5L (EQ-5D) is additionally employed quarterly as a generic measurement instrument that utilizes a standardized, preference-based procedure to ascertain the state of health.

Additionally, two unvalidated questionnaires are implemented to assess (1) patients’ well-being and satisfaction, particularly with regard to side effects, developed by patient representatives in the study team, and (2) information on marital status, household, place of residence (city/country), school-leaving qualification and occupation.

### Recruitment rationale and procedures

#### DigiNet intervention group

The recruitment is conducted by the participating, officially approved DigiNet sites within the two study regions, including nNGM centers and regional partners. nNGM uses a ‘broad consent’ for real-world data research; therefore, DigiNet patients require only a brief additional study consent, especially for the use of the patient portal. DigiNet consent can be obtained at the same time as nNGM consent or after receiving an nNGM diagnostics report, provided that all DigiNet clinical inclusion criteria are met. Additionally, depending on the statutory health insurance provider, the DigiNet intervention group requires a specific ‘Declaration of Participation’ (Teilnahmeerklärung) for the study-related Special Care Contracts under § 140a SGB V. These contracts facilitate the transfer of claims data from participating statutory health insurers for the purpose of DigiNet evaluation. Participation is entirely voluntary and patients can withdraw their consent to participate in the study at any time without stating reasons.

#### Population-based control group

The population-based control group will be identified by matching cohorts within the state cancer registries involved in the study once active recruitment and follow-up of the DigiNet intervention group is complete. The reason for generating the population-based control group through cohort matching within the cancer registries is due to specific stand-alone characteristics of the German cancer registration. In April 2013, the Cancer Early Detection and Registry Act (Krebsfrüherkennungs- und -registergesetz, KFRG) was passed in Germany, establishing a nationwide legal basis for the operation of clinical cancer registries across all German federal states (Möslein et al. 2013). The primary aim of clinical cancer registries is to ensure the quality of care for cancer patients. Therefore, by law, all physicians in Germany who diagnose or treat tumor diseases are obliged to report such cases to the state cancer registry using a standardized data format (oBDS), that precisely defines the parameters to be recorded. No patient consent is required. Data are collected from the diagnosis, through every step of treatment and follow-up, to relapses, survival, and death. In 2014, data completeness in the German cancer registration was > 90% for 12 of the 16 state cancer registries (Arndt et al. 2020). Therefore, the cancer registries in the model region represent the entire population of diagnosed stage IV NSCLC patients and, after excluding patients of the DigiNet intervention group and nNGM control group, comprise a suitable control group.

### Outcomes

**Fig. 1:**
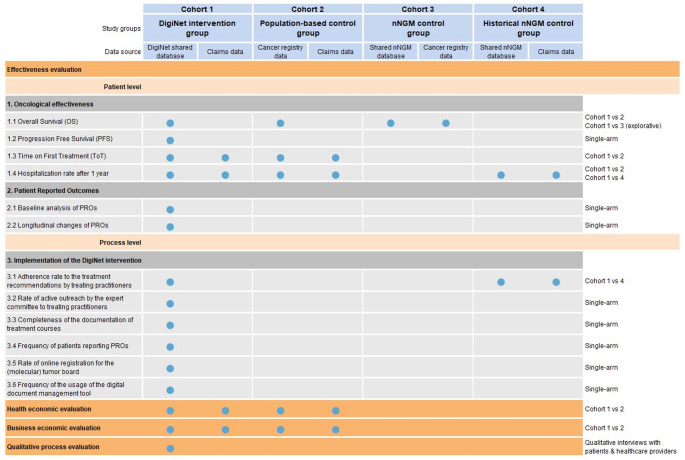
Overview of the data sources, cohorts, and endpoints of the different core areas of the DigiNet evaluation

The evaluation of the DigiNet study is divided into four core areas, which address different objectives with different outcome measures. An overview of the outcomes, the respective data sources, and study groups is provided in Fig. 1. All outcomes will be analyzed during the evaluation phase of the study.

#### Effectiveness evaluation—primary outcome

The primary endpoint is the overall survival (OS) in months, comparing the DigiNet intervention group and the population-based control group. Additionally, OS between the DigiNet intervention group and the nNGM control group will be compared as sensitivity analysis.

#### Effectiveness evaluation—secondary outcomes

Secondary endpoints comprise the time on first-line treatment in months and the hospitalization rate after one year (in %), comparing the DigiNet intervention group and the population-based control group. Progression-free survival and PROs are analyzed only in the DigiNet intervention group (no control group).

#### Process evaluation

The process evaluation is performed solely for the DigiNet intervention group. Process measures comprise the adherence rate to the treatment recommendations by treating physicians, the rate of active outreach by the expert committee (lung cancer specialists) to treating physicians in the treatment course, the completeness of the documentation of treatment courses, the frequency of patients reporting PROs, the rate of registration of DigiNet patients for the (molecular) tumor board and the frequency of the usage of the digital document management tool. All process measures are reported in percent.

In addition to the quantitative process evaluation, a qualitative process evaluation (formative evaluation) is carried out to identify facilitating and inhibiting factors as implementation determinants. Qualitative interviews with healthcare providers and patients will provide a comprehensive understanding of the modes of action of the complex intervention, and insights will be gained into the changes achieved by DigiNet during the therapy process. In order to ascertain the practicability, acceptance, and personal benefit assessment of PROs, a detailed analysis will be conducted. Applying a semi-structured guide based on a literature search, the interviews will be conducted over the entire study duration to account for possible changes in perspective.

#### Health economic evaluation and economic evaluation

The objective of the health economic evaluation is to address the question of cost-effectiveness of the DigiNet intervention, specifically comparing the financial implications of participation in DigiNet with those in the population-based control group (non-DigiNet and non-nNGM patients). To this end, the economic analysis will compare the costs of DigiNet versus usual care over a 12-month period from the perspective of the statutory health insurances (SHI).

Additionally, the business evaluation will assess the process and implementation costs from the perspective of healthcare providers. At the process level, the focus is on evaluating the implementation of DigiNet as a new healthcare model in routine care to identify factors that facilitate or hinder its implementation, particularly with regard to financial consequences for healthcare providers.

### Patient and public involvement

Active involvement of patient representatives in the DigiNet study is based on years of close partnership with the Zielgenau e. V. organization (www.zielgenau.org). Patients have been involved in the project application and the study protocol from the outset and form their own working group. They are actively involved in the project and contribute to aspects such as planning the collection of PROs, including frequency, layout, wording in patient-friendly language and the selection of validated instruments, as well as advising on the structure and content of the new patient portal. In addition, the patient working group developed its own PRO questionnaire, which will be analyzed independently. The public was not actively involved in the study.

### Sample size justification and power calculation

**Fig. 2:**
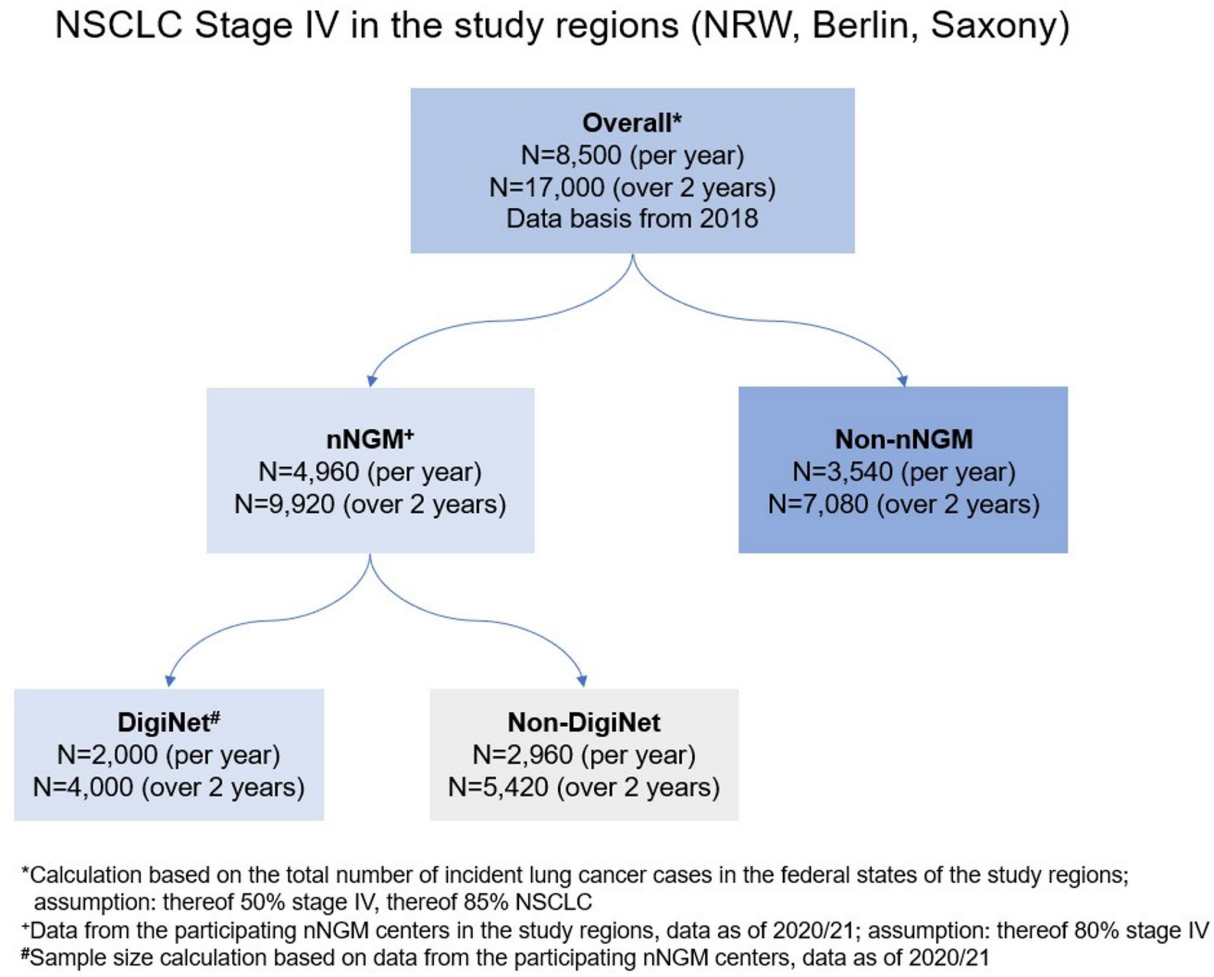
Sample size calculation and assumptions

According to the annual reports of the participating cancer registries, a total of around 20,000 people are diagnosed with lung cancer in the study regions each year (Berlin: N = 2597, NRW: N = 15,111, Saxony: N = 2383; data as of 2018). Approximately 50% of these cases are diagnosed at stage IV, of which 85% are NSCLC, resulting in 8500 patients with stage IV NSCLC annually (see Fig. 2). Each year, N = 6200 patients with advanced NSCLC (stage IIIB and IV) participate in the nNGM in the study regions (data provided by the nNGM office). The proportion of patients with stage IIIB and IV is approximately 20% and 80%, respectively. Accordingly, around N = 4960 patients with stage IV lung cancer are participating in the nNGM, which can be further subdivided into patients participating and not participating in DigiNet. The nNGM centers of the model regions, which participate in DigiNet, provided their sample sizes for 2020/2021. In these nNGM centers, an average of N = 1900 patients with stage IV lung cancer are participating in nNGM each year. In addition to further physicians who will participate in the nNGM and DigiNet, we expect N = 2000 potentially eligible patients with stage IV NSCLC in the study regions each year. The DigiNet recruitment phase will last just under two years (22 months), so the sample size will be doubled accordingly. Over the entire recruitment period, a total of N = 7080 patients will remain in the population-based control group (2), who will participate neither in the nNGM nor in DigiNet (see Fig. 2).

The objective was to demonstrate sufficient statistical power (> 80%) for the primary endpoint of overall survival (OS) over the 22-month recruitment period. Based on a study, which compared survival benefits between nNGM participants and routine care, a hazard ratio (HR) of 0.85 was used for the power calculation (Kästner et al. 2024). Assuming a mean follow-up of 1.5 years and a survival probability of 34.5% for the control group, the sample size of 850 DigiNet patients and 7,080 control group patients are expected to achieve a power of 89% with a 10% loss to follow-up (LTFU) rate, and 86% with a 20% LTFU rate. Thus, the sample size is sufficient to demonstrate longer OS for DigiNet participants compared to the control group. The power was calculated for a log-rank test with a type one error (α) of 5% using StataCorp. 2021.

### Data collection and secondary data sources

#### Shared DigiNet (and nNGM) database

After enrollment, patients will be regularly followed up as part of DigiNet, with treatment courses continuously documented in a shared digital database (NDP) by the DigiNet physician.

**Fig. 3:**
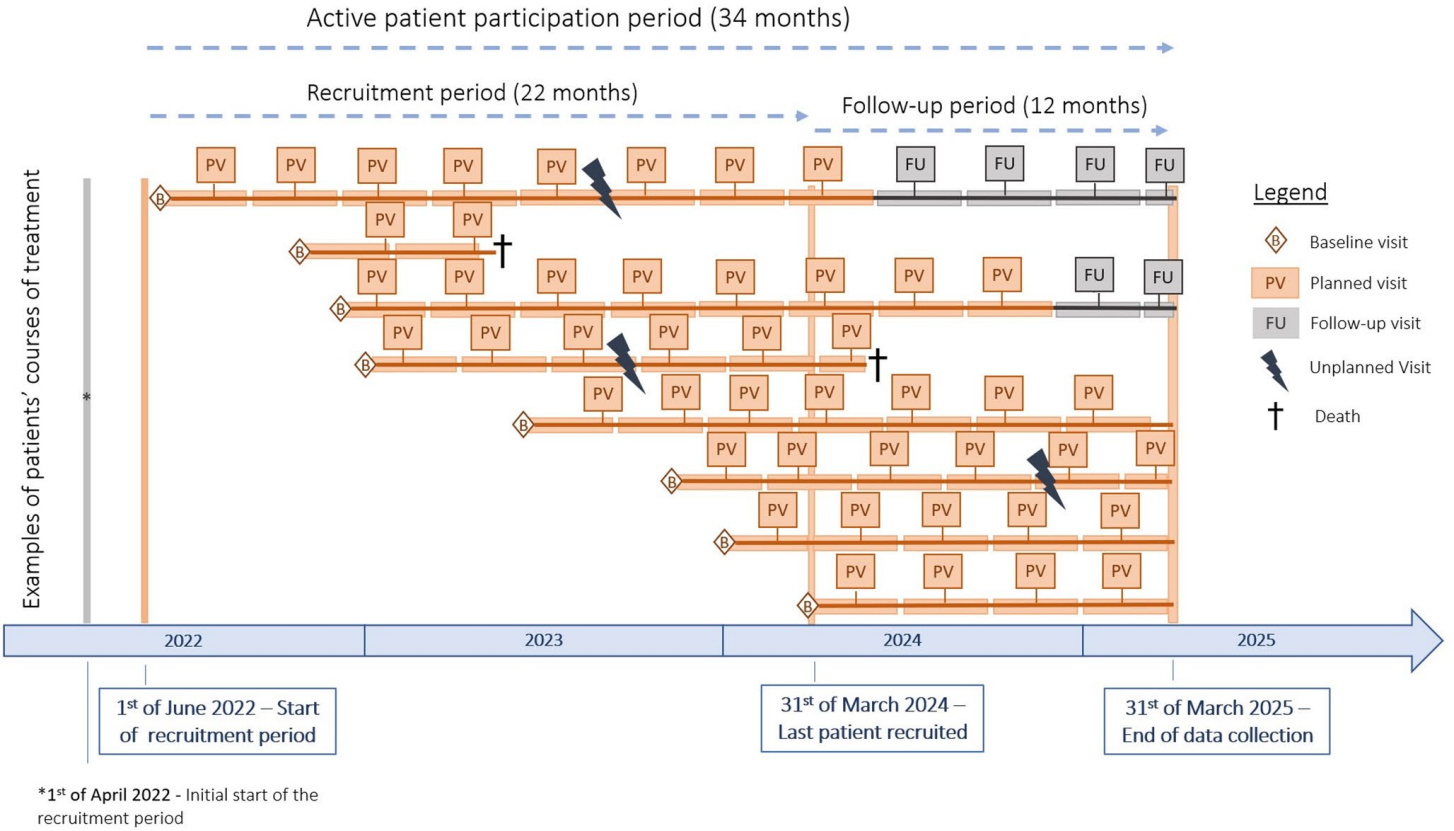
Schematic overview of the study’s active patient participation period and visit types

The baseline visit is documented immediately after molecular diagnostic results become available and therapy is initiated by the DigiNet physician. Collected data include nNGM molecular and pathological results (e.g., mutation status, MURIEL/MURIPEDIA codes, tumor stage, tumor grade, histology, and date of stage IV NSCLC diagnosis), prior oncological diagnoses, adherence to nNGM therapy information and its implementation, as well as the general health status assessed via the Eastern Cooperative Oncology Group performance status (ECOG-PS). Following the baseline visit, each patient undergoes four event cycles, each covering six months, resulting in a total active study period of up to two years. At least two visits per event cycle are expected, with additional visits conducted as needed. Visits are categorized as either planned or unplanned, depending on routine care requirements. Planned visits follow a regular schedule, whereas unplanned visits occur as unscheduled consultation of the patient due to illness or drug-related reasons (see Fig. 3). The primary difference in documentation is that unplanned visits include the reason for consultation, whereas planned visits do not.

Throughout both planned and unplanned visits, data are collected on systemic and interventional therapy courses (systemic and interventional therapy), treatment response, hospital stays, ECOG-PS, vital status, and PROs. After the two-year active study period, physicians may voluntarily document follow-up visits, which reflect planned visits in content but indicate that the active phase has concluded. For patients who withdraw their consent to participate in the study, the reasons are recorded in the NDP.

#### Patient-reported outcomes (PROs)

The PROs are assessed digitally by the patients themselves via the patient portal, which is integrated into the NDP, enabling secure and personalized data assessment. At recruitment, the patients are asked to provide their email address. The treating physician then documents the email address in the NDP, and the patient is given access to the patient portal and can complete the PRO questionnaires electronically. After recruitment to the DigiNet study, a baseline assessment of the PROs is performed. Subsequently, the PROs are obtained regularly every four weeks. To ensure compliance with regard to PRO participation, patients regularly receive automated email reminders. Additionally, treating physicians are encouraged to remind patients regularly to complete the PROs. In case of a change in therapy based on PRO results, the treating physician notes this decision in the NDP and documents the details of the treatment change.

During the course of the study, the option of paper-based PRO documentation was introduced in addition to the electronic PRO documentation (in March 2023). For patients choosing the paper option, the project team manually enters the paper-based questionnaires into the database.

#### Qualitative interviews and survey for healthcare providers

For the interviews, the recruitment of healthcare providers and patients is conducted through mail, email or personal contact. Two rounds of interviews are conducted: the first at the beginning of the recruitment process and the second after its end. These interviews with both healthcare providers and patients, are recorded following informed consent and later transcribed. During transcription, any identifying information such as names and locations is pseudonymized.

#### Cancer registry data

The State Cancer Registry of North Rhine-Westphalia, the Clinical Cancer Registry of Saxony, and the Clinical-Epidemiological Cancer Registry of Brandenburg-Berlin participate in DigiNet and provide data after approval of a data access request in accordance with the currently applicable use and access rules for the population-based control group, the nNGM control group and the DigiNet intervention group (for quality assurance purposes). The legal basis for the use of cancer registry data for research purposes are the respective cancer registry laws of the three federal states. The data are requested based on the oBDS and include sociodemographic variables such as age, sex, general performance status (ECOG-PS), and history of previous malignancies, as well as information on the NSCLC diagnosis, such as date of diagnosis, ICD-O-3 code (including morphology code and topography), tumor grading, clinical and pathological TNM classification, and presence and location of metastases. Additionally, therapy-related data is requested, covering information on surgery, residual tumor status, radiotherapy, and systemic therapies (including start and end dates as well as substances applied). Furthermore, information on patient outcomes, including tumor status over the course of the disease, as well as date and cause of death, is requested.

#### Claims data

In addition to the data from the state cancer registries in the model regions, data from the statutory health insurances participating in the DigiNet study (AOK Rheinland/Hamburg, AOK NordWest, AOK PLUS, AOK Bayern, AOK Nordost, BARMER, IKK classic, and Mobil Krankenkasse) are also required for the evaluation. The data set will include demographics, insurance periods, outpatient and inpatient data, medication, remedies and aids, care requirements, and incapacity for work. Moreover, costs associated with staff training and the delivery of the intervention will be quantified in order to assess the financial implications of the intervention. The legal basis for using the claims data for research purposes is § 75 of the Tenth Book of the German Social Code (SGB X) which regulates the transfer of claims data for research and planning. The provision of the claims data requires the approval of an application submitted to the German Federal Office for Social Security.

### Data linkage

The independent trusted third party (TTP) in Greifswald is responsible for merging the data from the various data sources (project database, state cancer registries, health insurance companies). Therefore, the TTP is divided into two technically, organizationally, and personnel-wise separate units. The unit “THS Greifswald” exclusively manages identifying data, whereas the unit “Data Merging” exclusively manages anonymized or pseudonymized medical data (e.g. pseudonymized data from the NDP). After completion of recruitment and follow-up and subsequent data merging, the data of the DigiNet intervention group, the population-based control group, and the nNGM control group will be provided to the evaluators by the TTP in anonymized or pseudonymized form.

### Statistical analysis plan

The detailed statistical analysis plans for the effectiveness, process, health economic, and business economic evaluations are provided in Supplementary Information file SI2.

### Data monitoring

To ensure high data quality, the assessed data in the NDP is continuously monitored for compliance with the inclusion criteria, plausibility, and data completeness (i.e., missing mandatory information) by the DigiNet data manager on a case-by-case basis (central data monitoring). In the event of inconsistencies, queries are sent electronically to the treating physicians or specialized nNGM centers via the NDP. The study sites are fully responsible for the accuracy of the documented data. No on-site data monitoring is provided. For the purpose of quality assurance for the evaluation, pseudonymized data exports from the NDP are regularly provided to the evaluators. After data preparation, the evaluators systematically check the data for plausibility and completeness. Any inconsistencies are reported to the DigiNet data manager, who sends queries to the physicians or nNGM centers via the NDP.

## Ethics and dissemination

The DigiNet study protocol was reviewed and approved by the Ethics Committee of the University Hospital of Cologne (21-1521). Written informed consent will be obtained from all eligible patients prior to study participation. The study is conducted in accordance with Good Clinical Practice (GCP) guidelines and with the principles of the Declaration of Helsinki. The results are disseminated through publications in peer-reviewed scientific journals and presentations at academic conferences. Moreover, the research findings will be made publicly available on the funder’s website, on the project website and via social media.

### Protocol modifications

All amendments to the protocol will be made according to GCP requirements and must be approved by the funder of this study and by the ethics committee of the University of Cologne.

The initial challenge in the design of the study was that due to the absence of comparable studies, the reliability of the assumed magnitude of the effect of the DigiNet intervention on survival (hazard ratio) was limited. Therefore, a recruitment rate of 80% of all potentially eligible cases in the study region was initially assumed necessary (i.e., a recruitment aim of N = 3200 patients in the DigiNet intervention group) with a median OS in the DigiNet intervention group of 2.0 years and 1.3 years in the population-based control group, which would have been associated with a power of 100%. Over the course of the study, however, it was found that the willingness of seriously ill patients to participate in the study was markedly lower than expected. Furthermore, the study by Kästner, Kron, van den Berg, et al. enabled the quantification of the survival effect, resulting in a more realistic sample size recalculation, which was fully approved by the project sponsor (Kästner et al. 2024).

## Discussion

The DigiNet study aims to demonstrate the potential value of a new, digitally supported care model in direct comparison to routine care and as an extension of the nNGM. This approach addresses one of the most critical challenges in healthcare, ensuring integrated, knowledge-generating patient care with continuous access to and utilization of data. The establishment of this care model builds on the extensive groundwork laid by the nNGM, particularly in interdisciplinary collaboration bridging inpatient and outpatient care sectors among multiple project partners.

The nNGM centers collaborate closely with over 500 regional partners from both inpatient and outpatient care sectors to jointly provide care for over 19,000 nNGM patients annually. The network structure is now in its third funding cycle, supported by the German Cancer Aid. Additionally, Special Care Contracts under § 140a of the Fifth Book of the German Social Code (SGB V) have been established with the most statutory health insurances to provide full reimbursement for next-generation sequencing (NGS) diagnostics, currently covering over 95% of the German population. Through close integration and continuous knowledge exchange among key stakeholders, nNGM has demonstrated a significant survival benefit for its patients compared to non-nNGM patients, as evidenced by an evaluation based on health insurance data and real-world data (Kästner et al. 2024). By fostering close collaboration among interdisciplinary treatment providers, nNGM enables the continuous evaluation of care and the generation of shared, high-complexity medical knowledge.

In the initial study design, study sites were limited to the nNGM centers in the study regions and those providers who had requested molecular diagnostics within the nNGM, thereby already being nNGM partners. During the DigiNet recruitment process, it quickly became evident that physician changes occurred frequently, particularly when a nNGM diagnostic request originated from a hospital, while subsequent treatment was provided in an outpatient setting. This necessitated an expansion of study sites and, consequently, an extension of nNGM partnerships, ultimately strengthening the nNGM as a whole. This adaptation was essential to ensure a seamless representation of patient journeys and maintain data completeness.

The International Committee of Medical Journal Editors (ICMJE) requires clinical trials to be registered in a publicly accessible registry prior to the enrollment of the first participant. In the present study, the six-month delay between the initiation of recruitment and trial registration resulted from procedural adjustments during the early recruitment phase, as described above. The DigiNet study protocol remained unchanged throughout this period.

All study sites are digitally interconnected via the NPD, enabling the digital processing of clinical workflows, such as requesting nNGM molecular diagnostics and molecular tumor board admissions. All DigiNet physicians can securely access, share, and utilize patient data from different sources within the NDP in an unblinded manner, provided that it relates to the treatment context. For analysis and research purposes, additional pseudonymization is applied. The quality and completeness of data in the DigiNet study are ensured through continuous and systematic data management. Due to resource constraints, on-site monitoring will not be performed, despite its potential importance as a quality-enhancing measure.

However, currently available data sources, such as specific clinical databases, cancer registries, claims data, have specific limitations regarding completeness and representativeness due to their individual utility. By directly and innovatively linking these data sources within the DigiNet study, additional value is expected to be generated. The technical data linkage is, in itself, highly novel for Germany, particularly in light of the stringent data protection regulations. A key innovation compared to the nNGM is the active involvement of patients as both project partners and study participants, granting them greater autonomy over their data via the patient portal. Patients will design and evaluate their supplementary questionnaires and have access to their nNGM data, including MURIEL/MURIPEDIA-based information.

Until the nationwide implementation and full functionality of the electronic patient record (ePA (*gesund.bund.de. Ein Service des Bundesministeriums für Gesundheit.Gesundheit Digital. Elektronische Patientenakte (ePA)*, 2025)), the DigiNet study serves as a blueprint for shared data utilization in healthcare and research, piloted in two study regions. Following a successful evaluation, this new digitally supported care model should be intended for nationwide implementation into routine care.

## Electronic supplementary material

Below is the link to the electronic supplementary material.

Supplementary Material 1

Supplementary Material 2

## Data Availability

No datasets were generated or analysed during the current study.
