# Supplementary material for: DigiNet: Optimizing personalized care for patients with stage IV non-small cell lung cancer (NSCLC) through a digitally connected provider network–analysis plan of a prospective multicenter cohort trial
Source: J Cancer Res Clin Oncol. 2025 Sep 9;151(9):244. doi: 10.1007/s00432-025-06275-x (PMC12420542; doi:10.1007/s00432-025-06275-x)
Supplement: Supplementary file 2 — Supplementary Material 2 [file 432_2025_6275_MOESM2_ESM.docx]

**Supplementary Information File SI2**

**Statistical Analysis Plan – Effectiveness evaluation**

In general, descriptive statistics for nominal variables will be presented as frequencies (number and percentage). Interval scaled or metric variables will be presented as mean and standard deviation (SD) or median and interquartile range (IQR). Unless otherwise indicated, missing values will be listed as a separate category. For all statistical analyses, a two-tailed significance level (type 1 error) of *p* < 0.05 is set. Furthermore, a 95 % confidence interval (CI) will be reported in addition to the effect estimates. The statistical analyses will be primarily performed in R Studio. Other statistical programs that will be used for selected analyses are Python, SPSS, and SAS.

*Baseline Analysis*

For the DigiNet intervention group and the population-based control group, the timepoint of the (first) diagnosis of stage IV NSCLC is defined as baseline. The baseline evaluation will be performed as soon as the cancer registries provide the data of the population-based control group (planned from Q2/2025). It will be examined whether the two groups are comparable with regard to the distribution of baseline characteristics (age, sex, study region, ECOG-PS, and histology).

Due to the study design, there is a relevant risk of selection bias, whereby the intervention effect can be influenced by confounders. Therefore, the above-mentioned demographic and disease-related baseline characteristics are examined for differences between the DigiNet intervention group and the population-based control group. In the event of significant differences, propensity score matching or inverse probability of treatment weighting is performed to adjust the groups. However, the subsequent often substantial reduction in sample size, leading to a decrease in statistical power, must be critically considered. After performing propensity score matching, statistical power will be checked.

*Overall Survival Analysis*

The primary endpoint OS is compared between the DigiNet intervention group and the population-based control group in an intention-to-treat analysis. OS is defined as the time in months from diagnosis of stage IV NSCLC to death, regardless of cause of death. The observation period ranges between 12 and 34 months, depending on the time of recruitment. For patients who are still alive at the end of the study or whose survival status is unknown at the end of the study (drop-out or LTFU), survival is censored at the last known date at which the person was still alive.

For the analysis of the primary endpoint between the DigiNet intervention group and the population-based control group, the survival rates are first visualized graphically using Kaplan-Meier curves and compared using the log-rank test. The median survival for both study groups will be calculated together with the 95 % confidence interval. The hazard ratios and 95 % confidence intervals are then estimated for the main analysis using a multivariable Cox proportional hazards regression model after checking the model assumptions, whereby the following covariates with the following characteristics are considered: study group (DigiNet intervention group vs. population-based control group), age, sex (male vs. female), study region (North Rhine-Westphalia vs. Berlin vs. Saxony), ECOG-PS (0 to 4), initial diagnosis of NSCLC (yes vs. no) and histology (adenocarcinoma vs. squamous cell carcinoma vs. large cell carcinoma vs. other). Moreover, the OS of the DigiNet intervention group will be exploratively compared with an nNGM control group.

Sensitivity analyses will be performed to check the robustness of the results of the primary endpoint. In these analyses, OS will be compared between the DigiNet intervention group and the population-based control group, (1) considering cases only if they have survived for at least one month (landmark analysis), (2) including only patients who received systemic therapy, and (3) including DigiNet cases only if the time between diagnosis at stage IV and the date of written informed consent does not exceed one month. In addition, when multiple imputation is applied, a complete case analysis (analysis restricted to cases with complete data) is performed as sensitivity analysis for the primary endpoint.

*Progression-free survival analysis*

Progression-free survival (PFS) will be analyzed only in the DigiNet intervention group. No test for statistical significance will be performed. PFS will not be compared against the population-based control group, as it cannot be assumed that the progress of stage IV NSCLC patients is reliably and completely recorded in the participating cancer registries. However, data for the DigiNet intervention group will also be requested from the state cancer registries to verify this assumption. For the descriptive analysis of the PFS, the median PFS and the PFS rates at 6 months, 12 months, 18 months and 24 months with the corresponding 95 % confidence intervals will be presented and illustrated using Kaplan-Meier curves. In addition, the absolute frequencies of the events (deaths) and censored cases will be provided.

*Comparison of time on first-line treatment*

The time on first-line treatment is compared between the DigiNet intervention group and the population-based control group, based on claims data of the health insurances participating in the DigiNet study and cancer registry data. The time on first-line treatment (ToT, also referred to in the literature as Time to Treatment Discontinuation (Lasala et al., 2023; Walker et al., 2021)) is defined as the period from the first administration of a systemic oncological therapy after diagnosis of stage IV NSCLC to the complete discontinuation of first-line therapy, regardless of the cause. In this context, first-line therapy may consist of a single agent or a combination of several agents. In the case of a combination of several agents, first-line therapy is considered to end when all agents have been completely discontinued. In case of a change from mono- or combination chemotherapy to a targeted therapy within the first four weeks after diagnosis of stage IV NSCLC, the duration of first-line therapy starts with the administration of the targeted therapy. The rationale for this is that there are cases with high treatment pressure after diagnosis that require the immediate initiation of systemic therapy before the results of molecular pathology become available, and shortly thereafter, the subsequent therapy is adapted based on the molecular findings. This exception from the definition is adequate since the endpoint ToT is intended to reflect the efficacy and tolerability of first-line therapy, and a change of therapy in the presence of the molecular pathology findings is not related to the efficacy and tolerability of the first therapy. The comparison of the duration of first-line therapy (in months) between the two groups is performed using the unpaired *t*-test for two samples.

*Comparison of the hospitalization rate*

The hospitalization rate will be compared between the DigiNet intervention group and the population-based control group based on claims data of the health insurances participating in the DigiNet study. The hospitalization rate will also be compared between the DigiNet intervention group and the historical nNGM group. The hospitalization rate after one year will be analyzed by the Chi² test. Also, the mean frequency of hospitalizations within the first year after diagnosis and the number of hospital days in the first year will be compared between the two groups by the unpaired two-sample *t*-test.

*Patient-reported outcomes (PROs)*

The PRO questionnaires will be analyzed according to scoring algorithms provided by the developers. For the baseline analysis of PROs, the results of the PROs of the DigiNet patients will be presented descriptively overall and subdivided by therapeutic category (first-line therapy with TKI therapy, immunotherapy, or chemotherapy). In addition, the baseline characteristics of patients who have ever completed PROs (so-called responders) will be compared with patients who have never completed PROs during the study period (so-called non-responders). For further analyses, only responders are considered. Missing values for single items are handled according to the developer's specifications. The analysis of longitudinal changes will be displayed graphically, and influencing factors will be analyzed exploratively using mixed-effect models.

The questionnaire on patients' well-being and satisfaction, particularly concerning side effects, is evaluated by the patient’s representatives themselves. This is a key component of active patient participation in the DigiNet project.

*Missing Data*

In case of a high fraction of missing information in the covariates of the Cox proportional hazards model, multiple imputation (MI) of the missing values is carried out to analyze the primary endpoint if all requirements are met in order to counteract power losses. For this purpose, MI with chained equations (MICE) is performed with the respective covariates. The number of datasets to be imputed depends on the fraction of missing information (Madley-Dowd et al., 2019; White et al., 2011). The choice of the imputation model (linear, Poisson, logistic, etc.) depends on the structure of the respective variables to be imputed (van Buuren, 2018). The Nelson-Aalen estimator of the cumulative hazard of the survival time will be added to the imputation model (White & Royston, 2009).

**Statistical Analysis Plan – Process Evaluation**

The quantitative process analysis will be performed for the DigiNet intervention group only. The endpoint of the implementation of the nNGM therapy information will be compared between the DigiNet intervention group and the historical nNGM control group (Chi² test). Descriptive statistics for nominal variables will be presented as frequencies (number and percentage). Unless otherwise indicated, missing values will be listed as separate category.

The qualitative transcribed interviews are analyzed using qualitative content analysis according to Kuckartz and Rädiker (Kuckartz & Rädiker, 2024). Qualitative content analysis is a systematic method for evaluating qualitative data. It involves defining categories, creating a structured framework, and identifying and verifying relationships. Central to this analysis are categories that code material relevant to the research questions. Category formation can be deductive, inductive, or a combination of both. While primarily qualitative, the analysis may also incorporate quantitative evaluations and can focus on either categories or individual cases.

**Statistical Analysis Plan – Health Economic Evaluation**

The economic evaluation is conducted as a cost-effectiveness analysis.

*Data*

The resource utilization data will be collected from the baseline until the 12-months follow-up period, utilizing the claims data provided by the cooperating health insurances.

*Outcomes*

The primary outcome measure will be the overall survival. Costs will be measured by accumulated costs for health care use. Costs will be treated as continuous numeric values measured in Euros and Cents using claims data.

*Analysis population*

The full analysis set of the DigiNet intervention group will include all who provided informed consent to participate. A per-protocol set comprising all participants in the full analysis set for whom we assume no significant protocol violations (e.g., failure to receive all components of the intended intervention) may be conducted. The primary analysis will be conducted once the claims data for all patients have been received from their respective insurances and prepared.

*Statistical definitions and sensitivity analyses*

The data pertaining to cost and outcome (OS) will be integrated to facilitate the calculation of an incremental cost-effectiveness ratio (ICER). The non-parametric bootstrapping approach will be employed to ascertain the extent of sampling uncertainty surrounding the mean ICER. The mean differences in costs and net benefits between the treatment groups will be estimated, along with the associated 95 % confidence intervals. The initial analysis of the differences in overall mean costs between the groups will be conducted using appropriate statistical methods. Sensitivity analyses will be conducted for both the total costs and the specific cost categories (e.g., inpatient and outpatient treatment, medical devices, and pharmaceuticals).

*Subgroup analyses*

Analyses will be conducted on the final dataset to investigate how cost-effectiveness varies between patient subgroups (e.g., median age, sex). Any subgroup analyses for which the smaller subgroup includes fewer than 50 participants will be omitted.

*Data cleaning and missing data*

Face validity tests will be conducted on the data set to identify misspellings or anomalous outliers. Consequently, the unit supplying the data will be consulted to discuss the matter. Any corrections made will be duly documented in the SPSS or R code. The trial data will be subjected to a thorough examination for any instances of missing data. The most appropriate method for dealing with missing data will be determined based on two key factors: firstly, the proportion of missing data, and secondly, the likely mechanism of missingness. In the event of randomly missing data, appropriate imputation techniques may be employed. All corrections made will be documented in the SPSS or R code.

**Statistical Analysis Plan – Business Economic Evaluation**

The business economic evaluation focuses on process and implementation costs from the perspective of healthcare providers. Data collection is conducted synergistically as part of the qualitative process evaluation. The economic model is derived, among other sources, from claims data, billing records, reimbursement catalogs (such as uniform assessment standards (EBM)), and documentation from the DigiNet database systems. The total accumulated healthcare costs will be reported as continuous numeric values in Euros and Cents, derived from claims data, comparing the DigiNet intervention group and the population-based control group. Mean and median cost comparisons between the groups will be performed using appropriate statistical methods. As mentioned above, sensitivity analyses will also focus on total costs and specific cost categories.

Additionally, the costs of staff training and delivering the intervention will be assessed based on an eCRF questionnaire and qualitative interviews for the DigiNet intervention group. The cost of care for the control group is modeled using literature and expert opinions. Further analyses will explore the economic implications of the intervention.

**References**

Kuckartz, U., & Rädiker, S. (2024). *Fokussierte Interviewanalyse mit MAXQDA* (Vol. 2). Springer VS Wiesbaden. <https://doi.org/https://doi.org/10.1007/978-3-658-40212-9>

Lasala, R., Zovi, A., Isgrò, V., Romagnoli, A., Musicco, F., & Santoleri, F. (2023). Time to treatment discontinuation in first-line non-small cell lung carcinoma: an overview. *Current Medical Research and Opinion*, 1-10. <https://doi.org/10.1080/03007995.2023.2192610>

Madley-Dowd, P., Hughes, R., Tilling, K., & Heron, J. (2019). The proportion of missing data should not be used to guide decisions on multiple imputation. *J Clin Epidemiol*, *110*, 63-73. <https://doi.org/10.1016/j.jclinepi.2019.02.016>

van Buuren, S. (2018). *Flexible Imputation of Missing Data, Second Edition* Chapman and Hall/CRC. <https://doi.org/https://doi.org/10.1201/9780429492259>

Walker, B., Boyd, M., Aguilar, K., Davies, K., Espirito, J., Frytak, J., & Robert, N. (2021). Comparisons of Real-World Time-to-Event End Points in Oncology Research. *JCO Clinical Cancer Informatics*(5), 45-46. <https://doi.org/10.1200/cci.20.00125>

White, I. R., & Royston, P. (2009). Imputing missing covariate values for the Cox model. *Stat Med*, *28*(15), 1982-1998. <https://doi.org/10.1002/sim.3618>

White, I. R., Royston, P., & Wood, A. M. (2011). Multiple imputation using chained equations: Issues and guidance for practice. *Stat Med*, *30*(4), 377-399. <https://doi.org/10.1002/sim.4067>
